# Relationship between ferroptosis and healing of diabetic foot ulcer: a prospective clinical study

**DOI:** 10.3389/fendo.2024.1412373

**Published:** 2025-01-14

**Authors:** Qian Jiang, Long Tang, Shuao Xiao

**Affiliations:** ^1^ Department of Geriatrics, Second People’s Hospital of Gansu Province, Lanzhou, China; ^2^ Department of Burns and Plastic Surgery, Second Affiliated Hospital of Air Force Medical University, Xi’an, China; ^3^ Department of Plastic Surgery, the First Affiliated Hospital of Air Force Medical University, Xi’an, China

**Keywords:** ferroptosis, diabetic foot ulcer, wound healing, wound monitoring, chronic wound healing

## Abstract

**Objective:**

Diabetic foot ulcer (DFU) is one of the common complications in patients with diabetes mellitus (DM). In order to find a method to monitor and treat the refractory DFU, the ferroptosis level in DFU and traumatic wounds (TW) was monitored and the difference between them was analyzed. At the same time, this study further analyzed the correlation of ferroptosis levels with DM severity and DFU’s healing.

**Methods:**

A prospective cohort study was from January, 2021 to December, 2023 in the Second People’s Hospital of Gansu province, which included 59 patients with DFU and 42 patients with TW. We then used the kit to detect the indicators related to ferroptosis, including 4-Hydroxynonenal (4-HNE), Malondialdehyde (MDA) and reactive oxygen species (ROS), in the wound exudate of the two groups of patients.

**Results:**

The DFU group had higher ferroptosis level than the TW group (4-HNE: *P* = 0.003, MDA: *P*<0.001, ROS: *P*<0.001). The severity of diabetes was significantly associated with ferroptosis level in DFU patients(*r* = 0.936, *P* <0.001). The results of multiple regression analysis showed that 4-HNE (*β* = -0.182, *P* = 0.008), MDA (*β* = -0.478, *P* <0.001) and ROS (*β* = -0.394, *P*<0.001) significantly negatively predicted the healing rate of DFU.

**Conclusion:**

As a new monitoring and therapeutic target, ferroptosis level plays an important role in predicting the healing rate of DFU and assisting clinical treatment decision-making.

## 1 Introduction

Diabetic foot ulcer (DFU) is one of the serious chronic complications of diabetes. It is the main cause of disability and death in diabetes patients and brings heavy burden to the society (1). According to the definition of the international working group on diabetic feet, DFU is the foot ulcer in person with currently or previously diagnosed diabetes mellitus (DM) and usually accompanied by neuropathy and/or peripheral artery disease in the lower extremity (2). DFU is found in 19% to 30% of the world’s DM patients (3). Unlike other ulcers, DFU carries a high risk of amputation. It is estimated that on average every 20 seconds one diabetic suffers amputation due to DFU (4). The average patient spends more than $8,000 a year on DFU-related care, five times more than patients without foot ulcers (5). At the same time, unmeasured intangible costs have a significant impact on patients’ lives, including the costs of anxiety, depression, discomfort, pain, loss of independence, and other low quality of life (6). The effect of DFU on quality of life was comparable to that of breast cancer and recent myocardial infarction (7).

At present, DFU with infection and necrosis are usually treated by debridement to remove necrotic tissue, combined with glucose reduction, antibacterial and negative pressure drainage. Although some progress has been made in the treatment of DFU, there are still some patients with DFU failure to heal, eventually leading to amputation and even death. Based on the above problems, scholars of various countries are actively looking for the causes and mechanisms of DFU hard to heal. At present, there are several mainstream views on the causes of DFU, including chronic inflammation, infection, hyperoxidative stress, microcirculation disturbance, and accumulation of advanced glycation end products (AGE).

Dixon et al. identified a form of regulatory cell death distinct from apoptosis, necrosis, and other well-characterized cell death that was prevented by iron chelators and lipophilic antioxidants but not by apoptosis inhibitors, in 2012 (8). Therefore, they used the term ferroptosis to describe this iron-dependent, lipid peroxide-accumulating, nonapoptotic form of regulatory cell death. There were two main characteristics of ferroptosis: (1) in terms of cell morphology, ferroptosis resulted in cell mitochondria becoming smaller, membrane density increasing, cristae decreasing, and morphological changes in the nucleus not obvious; (2) In terms of cellular components, ferroptosis was manifested as the accumulation of lipid peroxides and the increase of reactive oxygen species (ROS) levels (9).

Recently, the relationship between ferroptosis and diabetic wound healing has received much attention. Cui et al. proposed that secretory autophagosomes (SAP) carrying cytoplasmic cargo reduce ferroptosis by decreasing generation and increasing discharge of free Fe^2+^ in skin repair cells, thereby accelerating wound healing in diabetes (10). Yu et al. reported that hesperidin, a naturally occurring flavonoid in citrus fruits, inhibits ferroptosis by activating SIRT3 and promotes diabetic wound healing (11). Although these studies have demonstrated a strong association between ferroptosis and diabetic wound healing, no clinical evidence has emerged in this area.

To this end, we conducted a prospective cohort study that measured the level of ferroptosis in DFU versus traumatic wounds (TW) and analyzed the correlation of ferroptosis with DM severity and ulcer healing.

## 2 Subjects and methods

### 2.1 Subject

This study is a prospective study, including 59 patients with DFU and 42 patients with TW who were hospitalized at the Second People’s Hospital of Gansu Province from January 2021 to December 2023. Inclusion criteria of patients with DFU: ≥18 years old; Wagner grade I - III; no surgical treatment before sampling. Inclusion criteria of patients with TW: ≥18 years old; injury caused by physical factors. The exclusion criteria of the two groups were: having other ischemic diseases; having serious diseases such as malignant tumor and tuberculosis; using enteral and parenteral nutrition for a long time; having mental illness or disturbance of consciousness; refusing to participate in this study.

### 2.2 Reagents

Lipid Peroxidation (4-Hydroxynonenal, 4-HNE) Assay Kit, Abcam, USA (ab238538); Lipid Peroxidation (Malondialdehyde, MDA) Assay Kit, Abcam, USA (ab118970); Tissue reactive oxygen species (ROS) test kit (DHE), baiaobolai, China (HR8821); Protease and Phosphatase Inhibitor; Abcam, USA(ab201119).

### 2.3 Data collection

After admission, the general information of the patients, including gender, age, body mass index (BMI), smoking status, alcohol consumption, blood pressure, blood glucose and blood lipids, were collected. Blood pressure (BP) includes systolic blood pressure and diastolic blood pressure; blood glucose includes fasting blood glucose (FBG), hemoglobin A1c (HbA1c) and oral glucose tolerance test for 2h’s blood glucose (OGTT(2h)); and blood lipids include triglycerides (TG) and total cholesterol (TC). At the same time, the exudation from the patient’s wound was collected. The specific process is as follows: remove the wound dressing, wash the four sides of the wound with normal saline, absorb the wound surface exudation with a syringe (1 mL), place it in a centrifuge tube (1.5 mL), and save it for 4°C. The sample was centrifuged at 3000 r/min for 10min to remove small tissue debris from the sample and absorb the supernatant for testing. 4-HNE, MDA, and ROS contents were measured in the samples to be tested using the corresponding assay kit.

Finally, the information on the DFU healing of the patients was collected. Image information was collected for the patient’s DFO at admission and 30 days after injury. During filming, a ruler placed around the DFO facilitates the late area calculation. Pictures of DFU enter into Imaging J software to calculate the ulcer area at admission (S_0_) and at 30 days (S_30_). The rate of ulcer healing was calculated by the following formula. DFU patients were usually admitted several days after injury. However, there was no healing trend in DFU before hospitalization, and the DFU area collected at hospitalization was considered the initial ulcer area during statistical analysis.


$$healing\;rate\;of\;DFU=S_{0}-\frac{S_{30}}{S_{0}}$$

### 2.4 Statistical analysis

Data analysis was performed using SPSS 26.0 statistical software (IBM, Armonk, NY). Continuous variables with normal distribution were described by mean ± standard deviation, and categorical variables were described by frequency and percentage. Sex characteristics were tested by chi-square test and other subject characteristics by independent *t*-tests. To test whether there was any statistical difference in ferroptosis between the DFU group and the TW group, we used independent *t*-tests. To test the correlation between ferroptosis-related indicators and indicators of diabetes severity, a canonical correlation analysis was used. To examine the correlation between ferroptosis-related indicators and wound healing, we used multiple linear regression analysis.

### 2.5 Sample size estimation

According to the statistical analysis of 4-HNE, MDA and ROS contents in DFU group and TW group in the pre-experiment, *K* =1, α =0.05, β =0.9, Drop-out rate (DR) =10%, using power analysis and sample size (PASS) software (NCSS, Kaysville, Utah) to calculate the sample size of at least 42, 26 and 38 cases. That is, the sample size of both the final DFU group and the TW group required at least 42 cases.

## 3 Results

### 3.1 Subject characteristics

As shown in **Table 1**, 59 patients in DFU group and 42 patients in the TW group were included in this study. In DFU group, 25 men were included compared with 34 women, with a mean age of 63.542 ± 15.001 years. In TW group, 21 men and 21 women were included, with a mean age of 50.667 ± 18.678 years. Compared with patients with TW, patients with DFU had higher systolic BP (*P* =0.003), diastolic BP (*P<*0.001), GBG (*P<*0.001), HbA1c (*P* =0.006), OGTT (2 h) (*P<*0.001), TG (*P<*0.001), TC (*P<*0.001).

**Table 1:** Analysis of the basic characteristics of the research object.

Parameters	Diabetic foot ulcer group (n=59)	Traumatic wound group (n=42)	*P*
Male sex, n (%)	25.000 (42.37)	21.000 (50)	0.448
Age (years)	63.542 ± 15.001	50.667 ± 18.678	<0.001 ***
BMI (kg/m^2^)	28.606 ± 3.399	24.045 ± 3.433	<0.001 ***
Smoking(/d)	8.237 ± 5.624	9.381 ± 6.789	0.358
Drinking (ml/d)	235.186 ± 135.947	242.071 ± 126.418	0.797
Systolic BP (mmHg)	140.034 ± 24.450	128.119 ± 15.461	0.003 **
Diastolic BP (mmHg)	95.372 ± 9.099	86.881 ± 6.114	<0.001 ***
FBG (mmol/L)	10.723 ± 2.416	4.746 ± 0.548	<0.001 ***
HbA1c (mmol/L)	9.220 ± 0.859	4.960 ± 0.615	0.006 **
OGTT(2h) (mmol/L)	12.280 ± 0.990	6.216 ± 0.9333	<0.001 ***
TG (mmol/L)	2.214 ± 1.056	1.160 ± 0.446	<0.001 ***
TC (mmol/L)	5.025 ± 1.590	3.726 ± 0.602	<0.001 ***

**: P<0.01, ***: P<0.001.

### 3.2 Analysis of ferroptosis in DFU and TW

As shown in **Table 2**, 4-HNE content in DFU group was 4.470 μg/mL, which was higher than 3.76 μg/mL in the TW group, which was statistically significant (*P* =0.003). MDA content in DFU group was 5.665 nmol/mgprot, which was higher than 3.621 nmol/mgprot in TW group, which was statistically significant (*P<*0.001). ROS content in DFU group was 371.912 U/mL, higher than 272.375 U/mL in TW group, which was statistically significant (*P<*0.001).

**Table 2:** Analysis of ferroptosis in diabetic foot ulcers and traumatic wounds.

Ferroptosis-related Indicators	DFU group (n=59)	TW group (n=42)	*P*
4-HNE (μg/mL)	4.470 ± 1.311	3.765 ± 0.869	0.003 **
MDA (nmol/mgprot)	5.665 ± 1.197	3.621 ± 0.967	<0.001 ***
ROS (U/mL)	371.912 ± 107.545	272.375 ± 86.714	<0.001 ***

**: P<0.01, ***: P<0.001.

### 3.3 Canonical correlation analysis of indicators of diabetes severity and ferroptosis-related indicators

The correlation between indicators of diabetes severity and ferroptosis-related indicators showed that FBG had low correlation with MDA and ROS (r = 0.374, r = 0.499), and moderate correlation with 4-HNE (r = 0.633). HbA1c was highly correlated with 4-HNE (r = 0.856), with moderate correlation with MDA and ROS (r = 0.660, r = 0.794). OGTT(2 h) was highly correlated with 4-HNE (r = 0.823), with moderate correlation with MDA and ROS (r = 0.621, r = 0.611) (**Table 3**). The canonical correlation analysis of indicators of diabetes severity and ferroptosis-related indicators showed that Model 1 and Model 2 were significant in the three Model groups (*P<* 0.05) (**Table 4**). However, Model 1 had a strong correlation (r = 0.936), and Model 2 had a weak correlation (r = 0.380). Based on the correlation and significance, Model 1 was selected for subsequent studies. In Model 1, the severity of diabetes has the largest absolute coefficient of FBG (1.004), representing the largest contribution of FBG to the demonstration of diabetes severity. ferroptosis had the largest absolute coefficient of MDA (0.503), representing the largest contribution of MDA to the display of ferroptosis (As described in the following formula. *V* and *W* represent a composite indicator of diabetes severity and ferroptosis, respectively).

**Table 3:** The correlation of indicators of diabetes severity and ferroptosis-related indicators.

	Fasting blood glucose	Hemoglobin A1c	OGTT(2h)
4-HNE	0.633 ***	0.856 ***	0.823 ***
MDA	0.374 **	0.660 ***	0.621 ***
ROS	0.499 ***	0.794 ***	0.611 ***

**: P<0.01, ***: P<0.001.

**Table 4:** Canonical correlation analysis of diabetes severity and ferroptosis.

	Canonical correlation coefficient (*r*)	Eigenvalue	Wilks statistic	*F*	Molecular DF	Denominator DF	*P*
Model 1	0.936	7.015	0.104	22.053	9.000	129.139	<0.001***
Model 2	0.380	0.169	0.832	2.608	4.000	108.000	0.040*
Model 3	0.167	0.029	0.972	1.585	1.000	55.000	0.213

*: P<0.05, ***: P<0.001.

Formula:

*V* = - 1.004×*X1* - 0.068×*X2 +* 0.063×*X3*

*W* = - 0.218×*Y1* - 0.503×*Y2* - 0.444×*Y3*

(*X1*: Fasting blood glucose; *X2*: Hemoglobin A1c; *X3*: OGTT(2h); *Y1*: 4-HNE; *Y2*: MDA; *Y3*: ROS)

### 3.4 Multiple linear regression analysis of ferroptosis-related indicators and ulcer healing rate

Multiple linear regression analysis showed that the regression equation was significant, with *F* =87.992 and *P<*0.001. Among them, 4-HNE (β =−0.182, *P* =0.008), MDA (β =−0.478, *P<*0.001) and ROS (β =−0.394, *P<*0.001) significantly negatively predicted the healing rate of DFU (**Table 5**). These variables collectively explained 81.80% of the variation in training match satisfaction.

**Table 5:** Multiple linear regression analysis of ferroptosis related indicators and ulcer healing.

	*B*	*β*	*t*	*P*	*F*	After adjustment *R* ^2^
4-HNE	-0.042	-0.182	-2.771	0.008**	87.992***	0.818
MDA	-0.121	-0.478	-6.005	<0.001 ***		
ROS	-0.001	-0.394	-5.056	<0.001 ***		

**: P<0.01, ***: P<0.001.

## 4 Discussion

Diabetic foot ulcer, as one of the serious complications of diabetes, brings great pain and heavy economic burden to patients. The long-term hyperglycemic state of diabetic patients leads to lower extremity neuropathy, vascular lesions and immune dysfunction, greatly increasing the risk of foot ulcers and infections. In severe cases, amputation may occur (12). In-depth exploration of the pathogenesis of DFU and searching for effective treatment methods are of great significance. The phenomenon of ferroptosis has shown an important role in the occurrence and development of refractory diabetic wounds in the field of basic research, but there is currently no clinical data to support it. Therefore, in this study, we compared and analyzed DFU patients and patients with TW, focusing on exploring the relationship between ferroptosis-related indicators in DFU patients and the severity of diabetes and ulcer healing rate. We found that the DFU group had a higher level of ferroptosis. The ferroptosis level of DFU has a high correlation with the severity of diabetes and can significantly negatively predict the healing rate of DFU. These findings provide clinical support for in-depth understanding of the pathogenesis of DFU and also point out potential directions for clinical treatment.

We first conducted a comparative analysis of clinicopathological characteristics of the two groups. Compared with the TW group, patients in the DFU group were older, and metabolic indicators such as blood pressure, blood glucose, and blood lipid levels were significantly increased. The pathophysiological mechanism of diabetes can explain this phenomenon. The body of diabetic patients is in a long-term hyperglycemic state. On the one hand, it leads to protein glycosylation, affecting the synthesis and cross-linking of collagen and reducing the structural stability of the wound tissue (13). On the other hand, when the body is stimulated by high glucose, excessive reactive oxygen species are produced, exceeding the clearance capacity of the antioxidant system in the body, disrupting the balance between oxidation and antioxidant defense and affecting the normal physiological functions of the body, such as mitochondrial dysfunction (14). High blood glucose mediates pathological changes such as increased inflammatory mediators, pericyte degeneration, thickened basement membrane, endothelial hyperplasia, reduced prostacyclin synthesis, impaired vasodilation, and increased procoagulant markers in wounds by increasing the level of reactive oxygen species. These changes have a huge negative effect on the healing of DFU (15).

Ferroptosis is a newly discovered form of regulated cell death. In recent years, it has received increasing attention in the occurrence and development of refractory diabetic wounds. The reduction of iron ion levels and the dysregulation of iron-related gene expression in diabetic wounds have been reported in many studies. Exogenous iron supplementation can promote extracellular matrix deposition of type 1 and type 3 collagen (16). Moreover, inactivation of ferroportin in skin macrophages also leads to delayed wound healing, defective granulation tissue formation, and reduced formation of blood vessels and lymphatics (17). In addition to abnormal iron metabolism, excessive oxidation and lipid peroxidation are also often observed in diabetic wounds. For example, Li et al. found that compared with the control group, the levels of reactive oxygen species (ROS), malondialdehyde (MDA), and lipid peroxidation in fibroblasts and vascular endothelial cells exposed to high glucose conditions were significantly increased, and the cells showed reduced survival rate and impaired migration. In addition, *in vivo* and *in vitro* experiments have shown that ferroptosis is strongly induced in diabetic wounds (18). Furthermore, a large number of studies have shown that local application of ferroptosis inhibitors can promote angiogenesis and stem cell regeneration by inhibiting ferroptosis, thereby accelerating the healing of diabetic wounds (19–22). Therefore, it is necessary to conduct clinical research on ferroptosis in patients with DFU.

In subsequent analyses, we examined the differential expression of ferroptosis-related indicators in two groups of patients with wounds. Ferroptosis is characterized by the accumulation of iron-dependent lipid peroxidation and reactive oxygen species (ROS) (23). The biomarkers of lipid peroxidation include malondialdehyde (MDA) and 4-hydroxynonenal (4-HNE) (24). Consequently, we selected MDA, 4-HNE, and ROS as indices for evaluating ferroptosis. We observed significantly elevated levels of ferroptosis in patients with DFU, which aligns with reports in the foundational research on ferroptosis. Thereafter, we conducted a canonical correlation analysis to assess the severity of diabetes in relation to ferroptosis indicators. Canonical correlation analysis is a multivariate statistical method employed to evaluate the strength and direction of associations between two sets of variables (25). We opted for this methodology due to its capacity to account for the intricate interplay among multiple variables, enabling us to assess the overall correlation between these two sets of variables rather than evaluating the correlation between each variable individually. Our findings revealed that fasting blood glucose exhibited a moderate correlation with 4-HNE, while glycated hemoglobin and glucose tolerance tests both demonstrated a strong correlation with 4-HNE, and moderate correlations with MDA and ROS. This suggests that the severity of diabetes is closely associated with the levels of ferroptosis, with 4-HNE potentially being the most predictive indicator of diabetes severity. 4-HNE is a secondary and persistent product under conditions of oxidative stress in the body, rendering it a more reliable indicator of pathological oxidative stress than ROS (26). Numerous clinical studies have indicated that elevated 4-HNE due to diabetes can induce significant pathophysiological alterations in multiple organ systems, including the cardiovascular (27), nervous (28), gastrointestinal (29, 30), and musculoskeletal systems (31, 32). Therefore, the monitoring and intervention of 4-HNE may hold significant importance for predicting and improving the healing outcomes of DFU.

Following the outcomes of the canonical correlation analysis, we proceeded to employ multiple linear regression analysis to assess the predictive value of ferroptosis markers on the healing rate of DFU. Multiple linear regression is a statistical technique utilized to evaluate the impact of multiple independent variables on a single dependent variable (33). In this study, DFU healing rate was considered the dependent variable, while 4-HNE, MDA, and ROS were treated as independent variables. Through the multiple linear regression analysis, it was revealed that the regression equation was significant (F=87.992, P<0.001), indicating that these ferroptosis markers could significantly predict the healing rate of DFU. Collectively, the results from both the canonical correlation analysis and the multiple linear regression analysis suggest that the severity of diabetes is closely associated with the levels of ferroptosis, and ferroptosis levels can serve as predictive factors for DFU healing rates.

Our research is accompanied by a number of inherent limitations that must be recognized. Firstly, the study was conducted within a single institution with a limited sample size, which may hinder the broad applicability and generalizability of our results. Additionally, the age of the control group, consisting of trauma patients, was lower than that of the DFU cohort, and this age discrepancy could potentially skew the expression levels of the biomarkers in question, consequently impacting the predictive accuracy of the healing outcomes for DFU. Lastly, there may be unmeasured confounding variables at play, such as medication side effects and the sensitivity of detection methodologies, which might influence the expression of ferroptosis biomarkers and obscure their association with the healing process of DFU.

## 5 Conclusion

Our study has revealed that patients with DFU exhibit significantly higher levels of ferroptosis compared to those with traumatic wounds, and this level is negatively correlated with the severity of diabetes and the healing rate of foot ulcers. Ferroptosis-related indicators hold promise as novel monitoring and therapeutic targets for DFU.

## Data Availability

The raw data supporting the conclusions of this article will be made available by the authors, without undue reservation.
